# Exploring non-compliance in a cluster randomised feasibility study to inform the design of the phase III trial

**DOI:** 10.1186/1745-6215-16-S2-P11

**Published:** 2015-11-16

**Authors:** Lauren Bell, Vipul Jairath, Brennan C Kahan

**Affiliations:** 1 Pragmatic Clinical Trials Unit, Queen Mary University of London, London, UK; 2 Oxford Clinical Trials Research Unit, Oxford University Hospitals, Oxford, UK

## 

Patients with acute upper gastrointestinal bleeding are often given a red blood cell (RBC) transfusion when their haemoglobin (Hb) drops below a certain threshold, however the optimal threshold is unknown. TRIGGER (Transfusion in Gastrointestinal Bleeding, ISRCTN 85757829) was a cluster randomised feasibility trial which assessed the feasibility of implementing a transfusion policy on a hospital wide scale. The trial recruited 936 patients across six UK hospitals.

One of the key feasibility outcomes was to assess adherence to the transfusion policy. Maintaining high adherence levels in an emergency setting where patients are typically seen by many physicians across multiple departments in a short space of time can be challenging. We therefore evaluated the reasons for non-adherence in order to inform strategies to increase adherence rates in the planned phase III trial. We separated protocol deviations according to whether a transfusion was given when it should not have been, or when a transfusion should have been given but was not. We looked at whether protocol violations were influenced by factors such as baseline characteristics of the patient, their perceived risk of adverse outcomes, clinician preference, or because the patient had already experienced an adverse outcome during the trial. Based on the results of this analysis, we provide recommendations for strategies to reduce non-adherence in the main trial and our findings may have broader implications to inform randomised trials of transfusion strategies in other therapeutic areas.

